# Supplementary material for: Complementary cortical and thalamic contributions to cell type–specific striatal activity dynamics during movement
Source: Sci Adv. 2026 Jan 28;12(5):eaea3935. doi: 10.1126/sciadv.aea3935 (PMC12851035; doi:10.1126/sciadv.aea3935)
Supplement: Supplementary file 1 [file sciadv.aea3935_sm.pdf]

Science Advances
AAAS

Supplementary Materials for

Complementary cortical and thalamic contributions to cell type–specific striatal activity dynamics during movement

Enida Gjoni *et al.*

Corresponding author: Gal Mishne, gmishne@ucsd.edu; Takaki Komiyama, tkomiyama@ucsd.edu

Sci. Adv. **12**, eaea3935 (2026)
DOI: 10.1126/sciadv.aea3935

This PDF file includes:

Figs. S1 to S5
Table S1

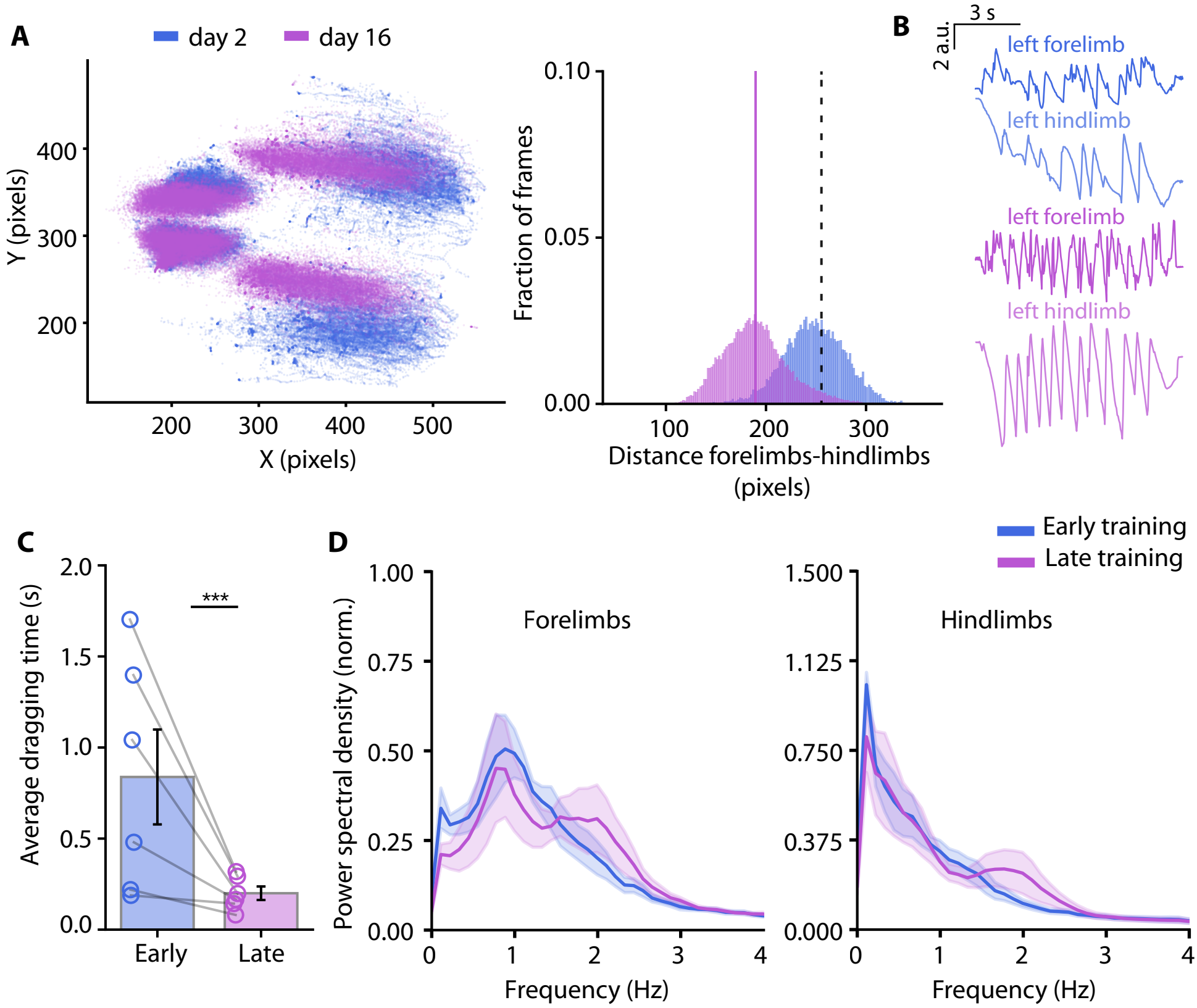
A
day 2
day 16
Y (pixels)
X (pixels)
400
300
200
200
300
400
500
Fraction of frames
0.10
0.05
0.00
100
200
300
Distance forelimbs-hindlimbs (pixels)
B
3 s
2 a.u.
left forelimb
left hindlimb
left forelimb
left hindlimb
C
Average dragging time (s)
2.0
1.5
1.0
0.5
0.0
***
Early
Late
D
Early training
Late training
Power spectral density (norm.)
1.00
0.75
0.50
0.25
0.00
Forelimbs
1.500
1.125
0.750
0.375
0.000
Hindlimbs
0
1
2
3
4
Frequency (Hz)
Frequency (Hz)

Fig. S1. Motor performance improves with training

A) Left, coordinates of the tracked limbs of an example mouse during an imaging session at day 2 and day 16 of training. Right, corresponding distributions of forelimb-hindlimb distances. Black line indicates mean of the day 16 distribution, dotted line indicates the threshold for dragging which corresponds to mean + 2*SD. **B)** Example trace of the first PCA component of the *x* coordinate of the left forelimb and hindlimb during one trial in an imaging session at day 2 and day 16 of training, from the same mouse. **C)** Average time spent dragging hindlimbs per trial (n = 6 mice) during early training (corresponds to days 2, 4, 6 of learning) and late training phase (days 12, 14, 16 of learning). Aligned rank transform mixed models: training day effect on dragging time, $p < 0.001$ (***); Bar plots correspond to the average across all mice, error bars indicate SEM. Linked datapoints correspond to the same mouse. **D)** Power spectral densities of the forelimbs (left) and hindlimbs (right) trajectories during early and late training, averaged across trials and animals (n = 6 mice). Aligned rank transform mixed models: forelimbs: training phase main effect, $p < 0.001$; frequency-band main effect, $p < 0.001$; training phase:frequency-band interaction, $p < 0.001$; hindlimbs: training phase main effect, $p = 0.166$; frequency-band main effect, $p < 0.001$; training phase:frequency-band interaction, $p < 0.001$; Traces indicate mean ± SEM.

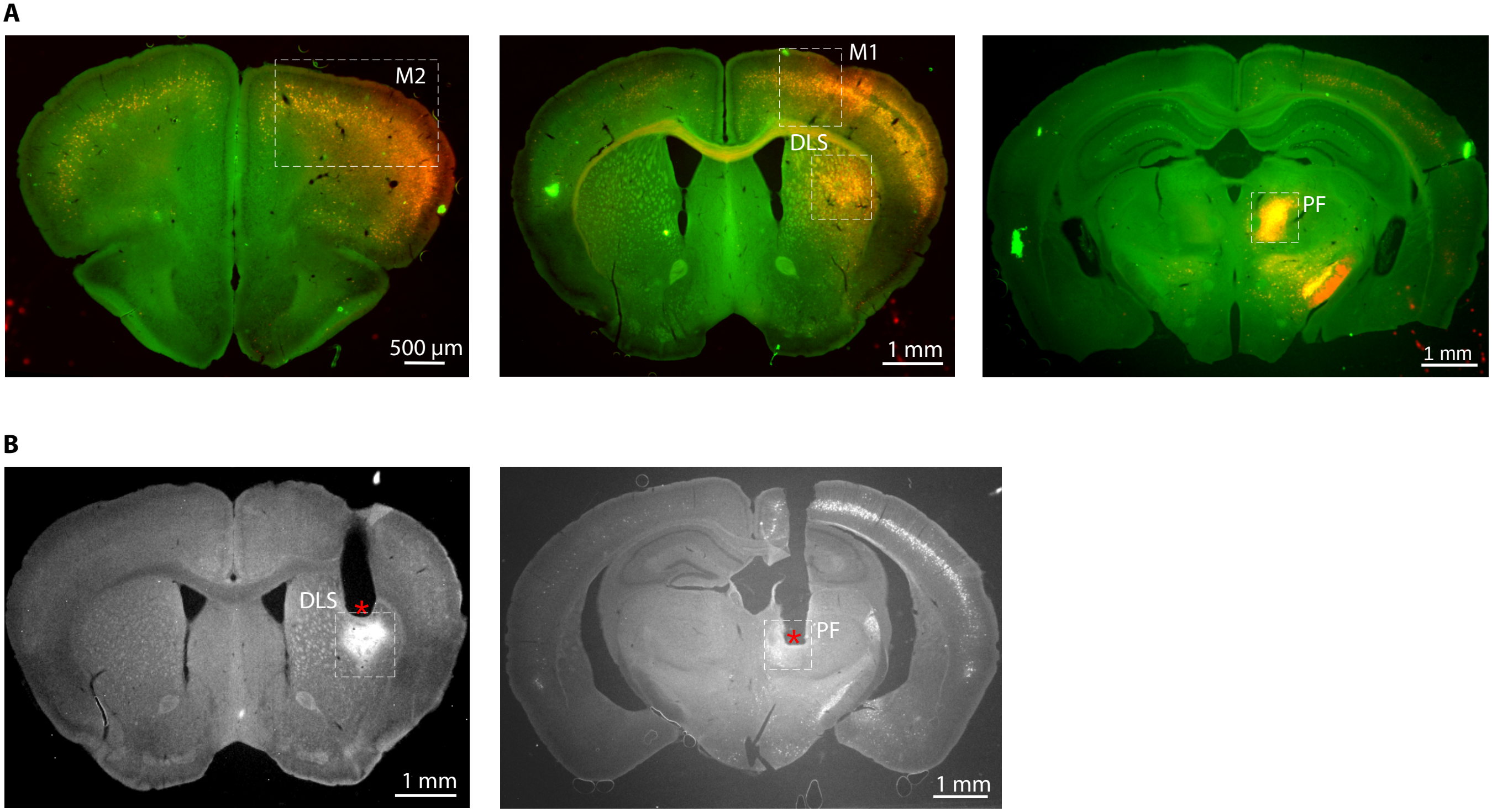

A
M2
500 µm
M1
DLS
1 mm
PF
1 mm
B
DLS
1 mm
PF
1 mm

Fig. S2. Labelling of input neurons in M1, M2 and PF via trans-synaptic spread of pseudo-typed rabies virus and GRIN lens targeting

A) Example coronal brain sections from a Drd1-Cre mouse that show the expression of mRuby-GCaMP6f (mRuby signal in red, GCaMP6f signal in green) via the EnvA-pseudotyped rabies in DLS (injection site) and in the three input regions imaged in this study: M1, M2 and PF. **B)** Example coronal brain sections that show the positioning of the GRIN lens in DLS (left) or in PF (right). Red asterisk indicates the GRIN lens bottom.

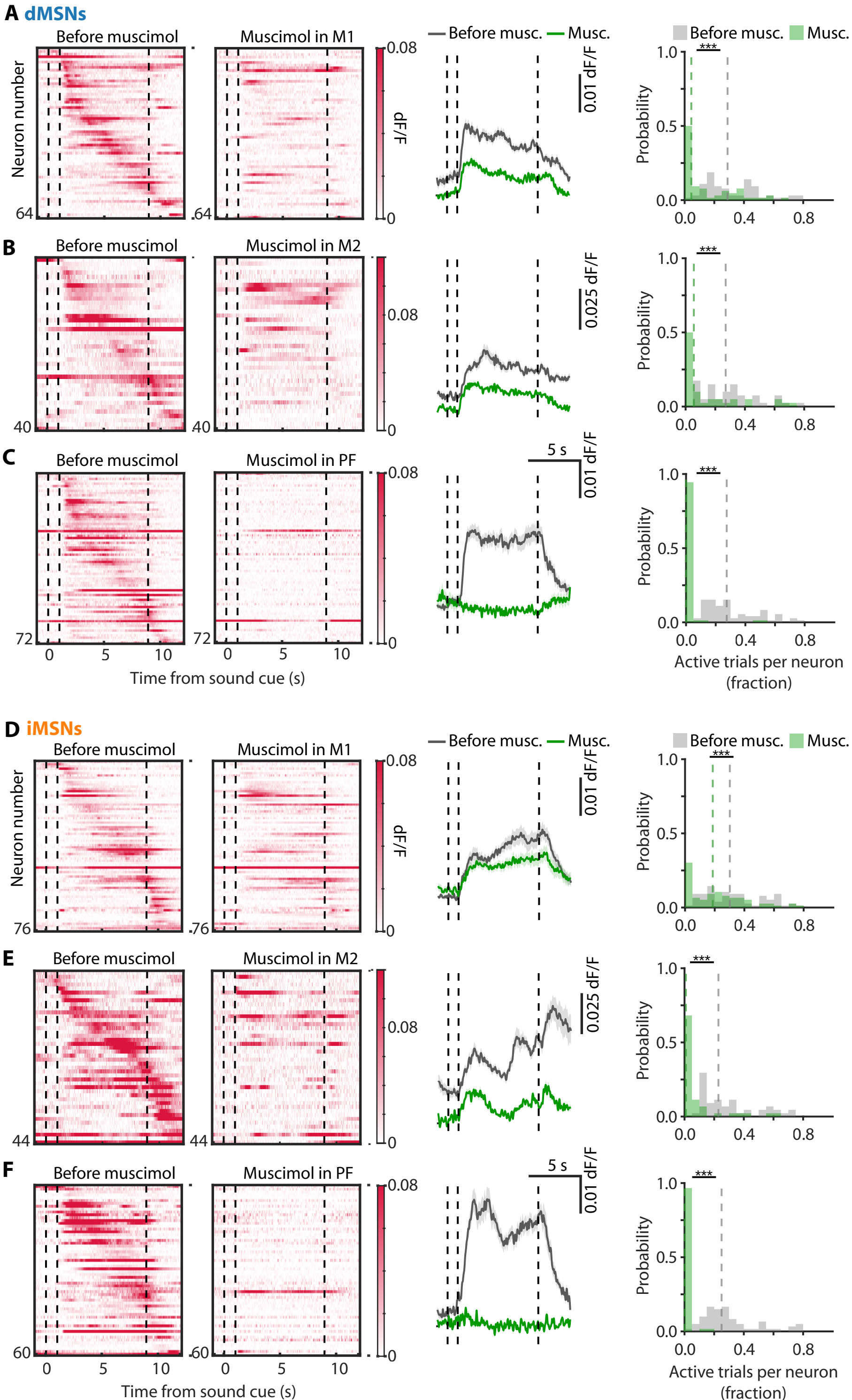

A dMSNs
Before muscimol
Muscimol in M1
Neuron number
64
0.08
dF/F
0
Before musc.
Musc.
0.01 dF/F
Probability
***
1.0
0.5
0.0
0.4
0.8
B
Muscimol in M2
40
0.025 dF/F
C
Muscimol in PF
72
5 s
Time from sound cue (s)
Active trials per neuron (fraction)
D iMSNs
76
E
44
F
60
0
5
10

Fig. S3. Muscimol inactivation of cortical or thalamic regions substantially reduces dMSN and iMSN activity

A) From left, trial-averaged activity of dMSNs from the same fields of view, imaged before and 30 minutes after muscimol injection into M1; population average activity of all dMSNs before and after muscimol injection into M1 (mean ± SEM); fraction of active trials in the imaged neurons before and after muscimol injection (n = 4 mice). **B)** Same as A) but for muscimol injections in M2 (n = 4 mice). **C)** Same as A-B) but for muscimol injections in PF (n = 5 mice). Black dotted lines indicate from left to right: sound cue, ladder onset, ladder offset. Gray dotted lines indicate medians. Traces correspond to mean ± SEM.

D-F) Same as A-C), but for iMSNs (muscimol injection in M1 (n = 4 mice); muscimol injection in M2 (n = 4 mice); muscimol injection in PF (n = 6 mice).

Aligned rank transform mixed models: muscimol effect on the fraction of active trials, $p < 0.001$ for all comparisons after vs. before muscimol injection (***).

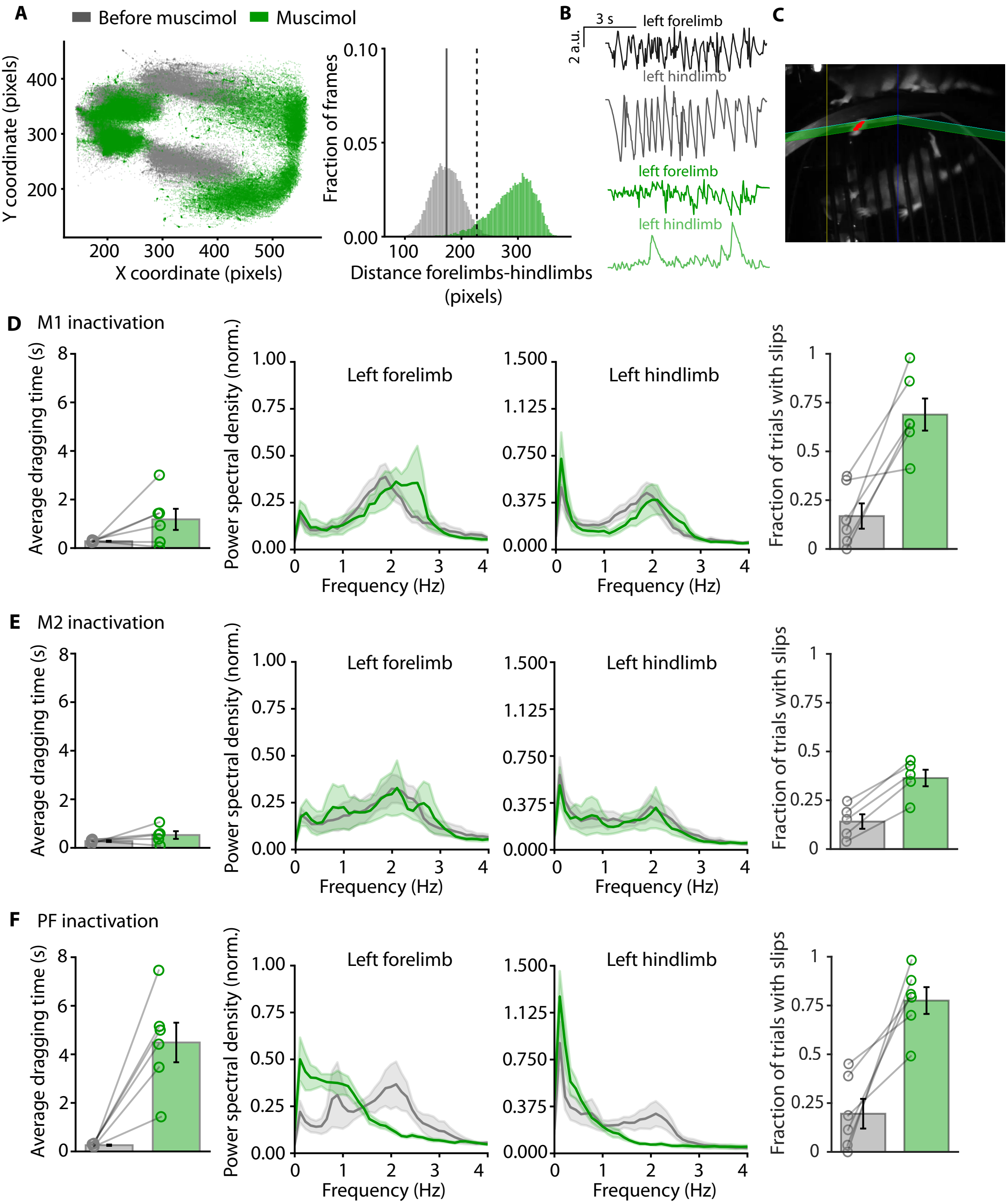
A
Before muscimol
Muscimol
Y coordinate (pixels)
X coordinate (pixels)
Fraction of frames
Distance forelimbs-hindlimbs (pixels)
B
3 s
2 a.u.
left forelimb
left hindlimb
left forelimb
left hindlimb
C
D
M1 inactivation
Average dragging time (s)
Power spectral density (norm.)
Left forelimb
Left hindlimb
Frequency (Hz)
Fraction of trials with slips
E
M2 inactivation
Average dragging time (s)
Power spectral density (norm.)
Left forelimb
Left hindlimb
Frequency (Hz)
Fraction of trials with slips
F
PF inactivation
Average dragging time (s)
Power spectral density (norm.)
Left forelimb
Left hindlimb
Frequency (Hz)
Fraction of trials with slips

Fig. S4. Muscimol inactivation of cortical or thalamic regions affects ladder task performance

A) Left, coordinates of the tracked limbs of a mouse during an imaging session before (gray) and after muscimol injection in PF (green). Right, corresponding distributions of forelimb-hindlimb distances during an imaging session before (gray) and after muscimol injection in PF (green). Black line indicates mean of the control session distribution, dotted line indicates the threshold for dragging which corresponds to mean + 2*SD. B) Example trace of the first PCA component of the *x* coordinate of the left forelimb and hindlimb during one trial in an imaging session before (gray) and after (green) muscimol injection in PF. C) Example video frame of a mouse on the motorized ladder and the forelimb slipping through the ladder rungs is marked in red. D) Effect of muscimol injection in M1 on motor performance (n = 6 mice). From left, average hindlimb dragging time per trial before (gray) and after (green) muscimol injection. Bar plots correspond to the average across all mice, error bars indicate SEM. Linked datapoints correspond to the same mouse. Muscimol session effect on dragging time, $p = 0.15$. Middle, power spectral densities of the forelimbs (left) and hindlimbs (right) trajectories before and after muscimol injection, averaged across trials and animals. Muscimol session effect on left forelimb $p < 0.001$, muscimol:frequency-band area interaction for left forelimb $p = 0.019$; muscimol session effect on left hindlimb $p < 0.001$, muscimol-frequency band interaction for left hindlimb $p < 0.001$. Right, fraction of trials with limb slips before and after muscimol injection. Bar plots correspond to the average across all mice, error bars indicate SEM. Linked datapoints correspond to the same mouse. Muscimol session effect on limb slips $p < 0.001$ (Aligned rank transform mixed models). E) Effect of muscimol injection in M2 on motor performance (n = 5 mice). From left, muscimol session effect on dragging time, $p = 0.36$; muscimol session effect on left forelimb $p = 0.474$, muscimol:frequency-band area interaction for left forelimb $p = 0.007$; muscimol session effect on left hindlimb $p = 0.089$, muscimol-frequency band interaction for left hindlimb $p = 0.415$, muscimol session effect on limb slips $p < 0.001$ (Aligned rank transform mixed models). F) Effect of muscimol injection in PF on motor performance (n = 6 mice). From left, muscimol session effect on dragging time, $p < 0.001$; muscimol session effect on left forelimb $p < 0.001$, muscimol-frequency band interaction for left forelimb $p < 0.001$; muscimol session effect on left hindlimb $p = 0.11$, muscimol:frequency-band area interaction for left hindlimb $p < 0.001$, muscimol session effect on limb slips $p < 0.001$ (Aligned rank transform mixed models).

**Model input**	**Classifier model**	**Classification accuracy ± SD (%)**
Trial-averaged neural activity	Linear SVM	53.87 ± 5.16
Ensembles of trials	Linear SVM	52.31 ± 6.53
Ensembles of trials	FCNN with attention	57.33 ± 3.86
Ensembles of trials	RNN with attention (TEA-net)	63.21 ± 3.98

Table S1. Comparison of cell-type classification performance of TEA-net vs. alternative models

Classification accuracy of dMSNs and iMSNs obtained by training different classifier models with trial-averaged neural activity or random ensembles of single trials. Classification accuracy was highest for TEA-net. Wilcoxon rank-sum test, TEA-net vs. SVM (trial-averaged data) $p = 0.004$; TEA-net vs. SVM (ensemble data) $p < 0.001$; TEA-net vs. FCNN $p = 0.011$. SVM: Support Vector Machine, FCNN: fully connected neural network, RNN: recurrent neural network, TEA-net: Trial Ensemble Attention Network.

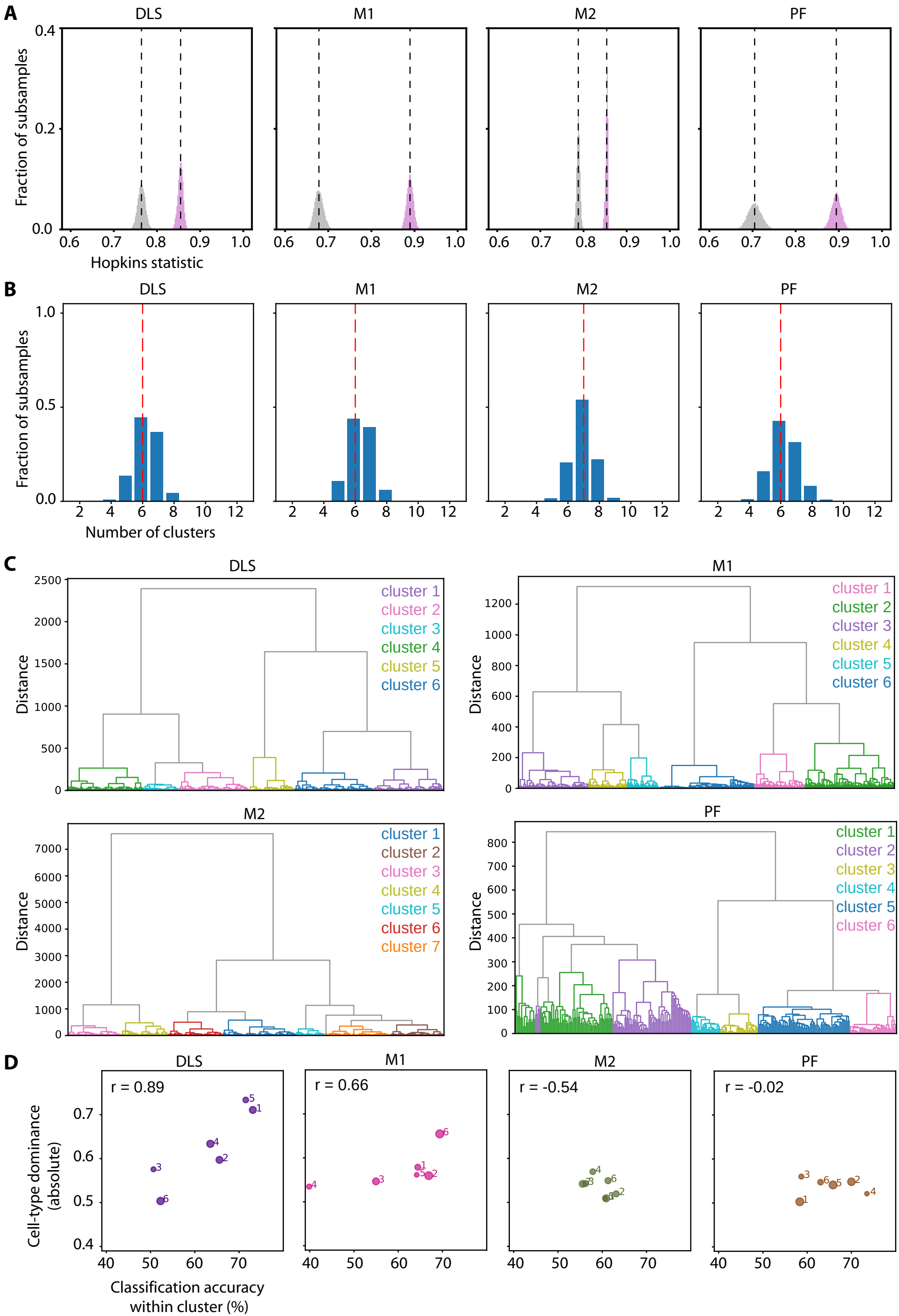

A
DLS
M1
M2
PF
Fraction of subsamples
Hopkins statistic
B
Fraction of subsamples
Number of clusters
C
Distance
cluster 1
cluster 2
cluster 3
cluster 4
cluster 5
cluster 6
cluster 7
D
r = 0.89
r = 0.66
r = -0.54
r = -0.02
Cell-type dominance (absolute)
Classification accuracy within cluster (%)

Fig. S5. Clusterability, optimal number of clusters and hierarchical-clustering dendrograms based on the latent representations for each region.

A) Hopkins statistic distributions of trial-averaged neural activity (in gray) and of the latent space representations (in violet) obtained from 10000 random subsamplings of 80% of the neurons in DLS, M1, M2 and PF. The latent representations yield consistently higher Hopkins values than the corresponding neural activity traces across all four regions, indicating that the latent space possesses a greater likelihood to form clusters. Hopkins statistic for latent representation and neural activity in each region, respectively (mean ± SD): DLS, 0.85 ± 0.01, 0.76 ± 0.01; M1: 0.89 ± 0.01, 0.68 ± 0.01; M2: 0.85 ± 0.002, 0.79 ± 0.003; PF: 0.89 ± 0.01, 0.71 ± 0.02. Dotted lines indicate the median of the distributions. **B)** Distributions of the number of clusters for the latent space representations, obtained from 10000 random subsamplings of 80% of the neurons. The mode of the distributions coincides with the number of clusters obtained when applying the Dynamic Tree cut approach to the complete datasets in DLS, M1, M2 and PF (red dotted line). **C)** Dendrograms obtained from the hierarchical clustering of the latent space representations of DLS, M1, M2 and PF neurons. Colors indicate the different clusters of neurons obtained in each region (see Fig. 3). **D)** Correlation coefficients between cell-type dominance and classification accuracy in each cluster, for each region. Circle size in each plot corresponds to the cluster size.
